# Drug-Induced Acute Interstitial Nephritis with Nifedipine

**DOI:** 10.1155/2016/1971465

**Published:** 2016-02-03

**Authors:** Léonard Golbin, Thibault Dolley-Hitze, Nolwenn Lorcy, Nathalie Rioux-Leclercq, Cécile Vigneau

**Affiliations:** ^1^CHU de Rennes, Service de Néphrologie, 35033 Rennes, France; ^2^CNRS UMR 6290, Équipe Kyca, 35043 Rennes, France; ^3^CHU de Rennes, Service d'Anatomie et Cytologie Pathologiques, 35033 Rennes, France

## Abstract

*Background*. Acute interstitial nephritis (AIN) is a frequent cause of Acute Kidney Injury (AKI). Drug hypersensitivity is the most common etiology and the list of drugs that can induce AIN is not exhaustive yet.* Case Report*. Here, we describe the case of a 43-year-old man who was treated with nifedipine (Adalate®) for Raynaud's syndrome. After nifedipine introduction, serum creatininemia progressively increased from 91 to 188 *μ*mol/L in a few months and AKI was diagnosed. Laboratory work-up results indicated the presence of tubular proteinuria and nonspecific inflammatory syndrome. Histological analysis found granulomatous interstitial nephropathy without necrosis in 20% of the kidney biopsy without immunofluorescent deposit. Nifedipine was stopped and corticosteroid treatment was started with a rapid but incomplete reduction of serum creatininemia level to 106 *μ*mol/L.* Conclusion*. This is the first case of AIN caused by nifedipine.

## 1. Introduction

The term acute interstitial nephritis (AIN) describes an immune-mediated kidney tubulointerstitial injury. AIN is a frequent cause of Acute Kidney Injury (AKI) and is observed in about 15% of patients hospitalized for acute renal failure [1, 2]. In most AIN cases, kidney injury is reversible, although in 30 to 70% of patients baseline renal function is not fully recovered [2].

AIN can be idiopathic or the result of drug hypersensitivity, infections, and immune-mediated diseases [3]. The first case of drug-induced AIN (DI-AIN) was described in 1968 and was caused by methicillin [4]. Drug hypersensitivity is now considered the most common cause of AIN. Many drugs have been involved, particularly antimicrobial agent, proton pump inhibitors, nonsteroid anti-inflammatory drugs [3, 5, 6], and fluindione [7]. Moreover, AIN could be an uncommon presentation of a commonly used medication, thus highlighting the risk of immune response to any medication. For instance, it has been reported that amlodipine besilate (a calcium channel blocker widely used as antihypertensive drug) can cause AIN [8]. Nifedipine (Adalate) is another calcium channel blocker that is used for the treatment of high blood pressure and also Raynaud's disease, Prinzmetal's angina, and angina pectoris (in association with beta-blockers). Previous studies reported that nifedipine can lead to AKI by causing deleterious kidney hemodynamic alterations [9].

We describe here the first biopsy-proven case of AIN caused by hypersensitivity to nifedipine.

## 2. Case Report

A 43-year-old Caucasian man was referred to the Rennes Hospital Nephrology Department to investigate an unexplained serum creatinine increase observed after the introduction of nifedipine for Raynaud's disease treatment. The patient's medical history included diabetes, secondary to a partial pancreatectomy following a work accident, treated with insulin since 2004, posttraumatic splenectomy, anxiety-depression syndrome, and family history of Raynaud's disease, but not of kidney disease, macroscopic hematuria, kidney stones, or urinary tract infections. A laboratory work-up performed on April 5, 2012, in the context of the Raynaud's disease follow-up showed that immunologic tests and C-reactive protein (CRP) were negative and serum creatinine concentration was 91 *μ*mol/L. His regular treatment included metformin, insulin, venlafaxine, clomipramine, and aripiprazole. Following the development of digital ulcers, nifedipine was introduced (on January 11, 2013) without any other treatment change. Within few weeks, serum creatininemia progressively increased to 169 *μ*mol/L (April 23) and 188 *μ*mol/L (May 29).

On June 11, 2013, the patient was hospitalized for renal investigations. Signs of urinary function alteration (with the exception of nocturnal pollakiuria), fever, and (present or past) extrarenal manifestations, especially cutaneous eruption and arthralgia, were not observed or reported. Body mass index was 26 kg/m^2^, and blood pressure was 125/70 mmHg. Physical examination found Raynaud's syndrome, but without active ulcers, cutaneous sclerosis, or trophic disorders. Cardiopulmonary auscultation and abdominal examination were normal. Urine dipstick test found protein ++, hemoglobin +, and leucocytes −. Urine electrophoresis showed proteinuria (0.41 g/g creatinine) of tubular origin (albuminuria/proteinuria ratio: 19%). The cytobacteriological urine test found 10^4^ red blood cells without leukocyturia and negative culture. Blood analysis showed no hydroelectrolyte imbalance, normal complete blood count (specifically no hypereosinophilia), moderate increase of inflammatory markers (CRP between 12 and 31 mg/L, polyclonal hypergammaglobulinemia), and normal albumin levels. Immunological tests (ANA, ANCA, and anti-GBM antibody) were negative; serum complement and angiotensin converting enzyme (51 UI/L) levels were normal. Blood culture, gastric aspirates for mycobacterium testing HVB, HVC, HIV, and CMV serology were negative, whereas anti-EBV antibodies suggested a previous exposure to this virus. Abdominal ultrasound and abdominal-pelvic computed tomography did not find any abnormality.

**Figure 1:**
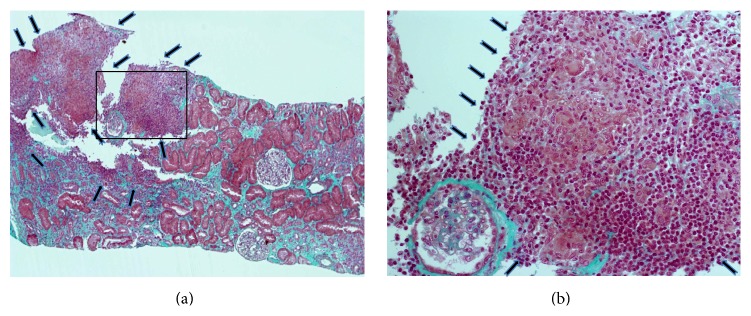
Renal biopsy during AKI. (a) Masson trichrome staining showing nonnecrotic interstitial infiltrate (arrows) affecting 20% of the kidney biopsy (magnification ×40). (b) Higher magnification of the area boxed in (a) showing the granulomatous interstitial infiltrate composed of epithelioid cells (arrow), some multinucleated giant cells (arrowhead), and a lymphoplasmacytic infiltrate (asterisk) (magnification ×200).

Histological analysis of the renal biopsy, performed on June 11, 2013, found nonnecrotic granulomatous interstitial nephropathy with epithelioid cells, some multinucleated giant cells, a lymphoplasmacytic infiltrate, and tubular atrophy in 20% of the kidney biopsy tissue (Figure 1). There was no immunofluorescent deposit and the Ziehl-Nielsen coloration was negative. Neither vascular nor glomeruli abnormality was detected.

Nifedipine was stopped on June 11 and corticosteroid treatment (0.5 mg/kg/day) was started on June 13. Serum creatininemia rapidly decreased from 175 *μ*mol/L (June 11) to 133 *μ*mol/L (July 12) and then remained stable (138 *μ*mol/L on October 4). The concentration of blood inflammatory markers decreased. Corticosteroids were progressively reduced and then stopped on October 4, 2013. Serum creatininemia further improved (115 *μ*mol/L January 24, 2014, and 106 *μ*mol/L on January 22, 2015).

## 3. Discussion

Here, we described a case of AKI caused by biopsy-proven nifedipine-induced AIN, an uncommon side effect of this widely used drug.

DI-AIN is due to an immune-allergic reaction to the concerned drug, as indicated by the clinical symptoms of hypersensitivity and presence of eosinophils, as well as its dose-independency and recurrence after reexposure to the same drug. The underlying mechanism could involve the development of an immune reaction (T cells and macrophages) after interstitial deposit of the drug and production of antibodies against the tubular basement membrane [5]. AIN clinical presentation is variable [3, 5] and the only consistent clinical feature is the acute or subacute kidney injury that classically appears 7–10 days after drug exposure. Indeed, the classic triad of hypersensitivity reaction (fever, cutaneous rash, and hypereosinophilia) is seen in less than 10–15% of patients with AIN. Nonspecific symptoms, such as digestive problems (nausea, vomiting), arthralgia, myalgia, flank pain, fever, chills, anorexia, and malaise, may also be observed. Urine dipstick testing often reveals proteinuria (1+ or 2+), while hematuria and leukocyturia are present in less than 50% of patients and hypereosinophilia in about one third of patients with DI-AIN. The presence of eosinophils in urine samples is suggestive of DI-AIN, but the test is not very sensitive [10]. Histological analysis, together with the clinical history, remains essential for DI-AIN diagnosis. In our patient with biopsy-proven AIN, we observed moderate extrarenal clinical inflammatory signs, without classic hypersensitivity reaction symptoms and without nonspecific clinical symptoms. The clinical presentation was evocative of nifedipine-induced AIN. However, the absence of any previously described case of nifedipine-induced AIN made the diagnosis more difficult, even in the absence of any other differential diagnosis. The short interval between the drug introduction and the serum creatininemia increase was strongly in favor of DI-AIN. Moreover, kidney function progressively improved after stopping the putative culprit drug and introducing corticosteroid therapy.

Concerning DI-AIN histological findings, nonspecific interstitial inflammation and tubulitis are classically found without glomerular or blood vessel lesions and with negative immunofluorescence. Moreover, mononucleate cells and lymphocytes are predominant in the interstitial infiltrate. These histological features can be associated with nonnecrotic granulomatous lesions, as observed in our patient [11].

Currently, there is no clear recommendation concerning DI-AIN treatment. The consensus is to stop the putative causative drug as soon as DI-AIN is suspected. Any delay in the drug withdrawal can adversely affect kidney function recovery. Different retrospective studies have evaluated the benefit of steroid therapy to limit the development of interstitial fibrosis and to facilitate the recovery of renal function, with controversial results [3, 5]. Clarkson et al. did not find any difference in the outcome (serum creatinine level) between patients with biopsy-proven AIN (*n* = 67) who received (60%) or did not receive corticosteroid therapy [12]. Conversely, González et al. found a beneficial effect of corticosteroids in patients with biopsy-proven DI-AIN (*n* = 61 of whom 52 received steroids) with a significant correlation between the delay in steroid treatment initiation and the final serum creatinine level [13]. The length of the steroid treatment also is debated.

Unfortunately, in the case of our patients, DI-AIN was diagnosed five months after the introduction of nifedipine. Therefore, the introduction of steroid therapy and nifedipine withdrawal resulted in a rapid but incomplete decrease of serum creatininemia. The long delay between nifedipine introduction and AKI diagnosis, leading to treatment interruption, could explain this partial recovery of kidney function. A recent prospective observational study reported that renal function often remains impaired after AIN [14].

This is the first biopsy-proven case of AIN induced by nifedipine. The list of drugs susceptible to cause DI-AIN is still increasing.
